# Rate of achievement of therapeutic outcomes and factors associated with control of non-communicable diseases in rural east Malaysia: implications for policy and practice

**DOI:** 10.1038/s41598-021-83168-2

**Published:** 2021-02-15

**Authors:** Zhi Yi Keng, Yu Mon Saw, Senk Chung Thung, Woon Wee Chong, Amanda Albert, Tetsuyoshi Kariya, Eko Yamamoto, Nobuyuki Hamajima

**Affiliations:** 1 Department of Internal Medicine, Hospital Umum Sarawak, Sarawak, Malaysia; 2 Department of Healthcare Administration, Nagoya University Graduate School of Medicine, Nagoya, Japan; 3 Nagoya University Asian Satellite Campuses Institute, Nagoya, Japan; 4 Department of Family Medicine, Hospital University Sains Malaysia, Kelantan, Malaysia; 5 Department of Emergency Medicine, Hospital University Sains Malaysia, Kelantan, Malaysia; 6 Department of Psychiatry, Hospital Sentosa, Sarawak, Malaysia

**Keywords:** Health care, Risk factors

## Abstract

Non-communicable diseases (NCDs) are an increasing problem worldwide, including in Malaysia. National surveys have been performed by the government but had poor coverage in east Malaysia, particularly in rural regions. This study aimed to describe the achievement of target therapeutic outcomes in the control of diabetes mellitus (DM), hypertension (HPT), and dyslipidemia (DLP) among diabetic patients in rural east Malaysia. A cross-sectional study was conducted among DM patients who visited the NCDs clinic in Lundu Hospital, Sarawak, Malaysia, from Jan to March 2016. In total, 214 patients (male, 37.9%; female, 62.1%) were recruited using a systemic sampling method. Multiple logistic regression models were applied to estimate the adjusted odds ratio (AOR) and confidence interval (CI) for the target therapeutic achievement in the control of DM, HPT, and DLP. Compared to the national average, therapeutic target achievement in Lundu was higher for DM (43.0% vs. 23.8%), equal for DLP (35.8% vs. 37.8%) but lower for HPT (30.9% vs. 47.9%). DM patients who had at least yearly HbA1c monitoring (AOR 2.30, 95% CI 1.04–5.06, *P* = 0.039), and those 58.7 years or older (AOR 2.50, 95% CI 1.32–4.74, *P* = 0.005) were more likely to achieve the therapeutic target for DM. Health promotion and public education regarding HPT needs to be emphasized in rural Malaysia. HbA1c monitoring at least once a year was one of the important factors associated with achieving DM control in rural east Malaysia. Accessibility to HbA1c tests and monitoring should be ensured for diabetic patients.

## Introduction

Non-communicable diseases (NCDs) are an increasing problem worldwide, including in Malaysia. The World Health Organization (WHO) reported that 71% of all deaths in 2018 were due to NCDs. Of these, cardiovascular diseases alone accounted for 44% of all NCDs deaths^1^. With rising population and prevalence for NCDs, health expenditures have been increasing globally^2^. Cost savings and the sustainability of the health system are major issues in nations that aim to achieve universal health coverage, thus, prevention and good control of NCDs have been shown to be the most economical way to manage the problem^3^. Malaysia government provides highly subsidised healthcare for all Malaysian and had achieved universal health coverage (UHC) in 1990’s^4^. As a tax funded national healthcare system however^4^, providing good quality and accessibility while managing cost is always a challenge.

Malaysia has seen a shift in the causes of mortality through the years. In 2007, for the first time, diseases of the circulatory system overtook infectious diseases as the number one cause of mortality in hospital settings and have continued to increase as a proportion of the total mortality^5^. The latest Ministry of Health Annual Report indicated a consistent disproportionate increase in nephrology and cardiology cases seen in specialist clinic from 2002 to 2011, increasing from 0.85% to 2.41% for nephrology and 1.01% to 4.58% for cardiology of all cases, both of which are highly dependent on the management of NCDs in the country^6,7^. The 5th National Health and Morbidity Survey (NHMS), performed in Malaysia in 2015, also reported that 17.5%, 30.3%, and 47.7% of adults have diabetes mellitus (DM), hypertension (HPT), and dyslipidemia (DLP), respectively, in Malaysia, a persistent increase since the first survey was conducted in 1986^8,9^.

Preventive health leads to an improvement of general public health, prevents overcrowding of tertiary centers, and reduces the long-term cost of healthcare^10^. Studies of NCDs have been conducted in Malaysia, although often concentrated only in west Malaysia and urban areas^11^. DiabCare 2013 was a multi-center study carried out to gauge the control of NCDs in DM patients in both east and west Malaysia; however, it was only performed in urban, tertiary centers^12^.

The rural population in Malaysia has been reducing through the years, but still made up 25% of the general population in 2017^13^. Rural regions are frequently plagued with infrastructure and resources restrictions thus may consist of a different pattern of control for NCDs compared to the urban region. However, information regarding rural primary healthcare centers’ achievements in NCDs are scarce.

To the best of our knowledge, factors affecting HbA1c achievement have been reported in studies in Malaysia but never in rural east Malaysia, where indigenous populations are predominant^14^. In addition, demography background such as the relationship between biochemical monitoring such as HbA1c and fasting lipid profile (FLP) to DM and DLP control have never been examined. The National Diabetes Registry Report (NDR) 2012 reported that up to 22.0% of diabetic patients managed in government clinics did not undergo any HbA1c monitoring in a year^15^. The ratio of HbA1c monitoring varies significantly between states, and is likely worse in east Malaysia, where many health facilities are not equipped with HbA1c-capable laboratories. Thus, this study aimed to examine the associations of the target therapeutic achievement in the three diseases with age, years of follow-up, sex, ethnicity, body mass index (BMI), smoking status, and relevant biochemical monitoring, and to describe the control of DM, HPT, and DLP in a rural setting in east Malaysia, bridging the gap of information available in Malaysia to assist policymakers in creating more effective long-term nationwide plans catering to both east and west Malaysia as well as urban and rural populations.

## Materials and methods

### Study design and participants

A cross-sectional study was conducted in Lundu District Hospital, in the northwest Kuching Division of Sarawak, east Malaysia. The population of Lundu constitutes 1.4% (33,413) of the total population of Sarawak. As comparison, the similarly sized neighbouring urban Kuching District have close to 19 times the number of population^16^. Lundu District population, like the rest of less developed Sarawak, generally engaged in agriculture, aquaculture, fishing as well as tourism as main economical activities^16^. The latest official document in 2010 combines both indigenous populations (mainly Bidayuh and Iban) and Malays in the statistics, making up 82.4% of the total population, while the Chinese population constitutes another 10.6%^17^. Besides an adequately maintained single carriageway dual lane highway connecting Lundu to other District, roads connecting to smaller villages in Lundu are mostly single lane, unpaved road, with wooden bridges connecting between multiple rivers running across the district, making even intra-district traveling challenging and time consuming.

Lundu Hospital offers both inpatient and outpatient services and is the sole healthcare center in the Lundu District. The hospital is a 40 bedded hospital with average of close to 130 outpatient visits per day. A DM patient is initially diagnosed in the outpatient clinic by a doctor and sent to register with the NCDs division under the outpatient department situated inside Lundu Hospital for further regular follow-ups. The patients’ history and treatment information are recorded in two places: 1) a record book given to patients to be brought around, if needed; 2) an identical diabetes registry record kept in the NCDs clinic as a precaution against lost information. Both records should receive identical input from the doctor upon follow-up, but due to errors and negligence, at times only one record receives the input.

Owing to the weaknesses mentioned above, this study was conducted by identifying patients and obtaining their record card during their follow-ups instead of merely extracting information from the diabetes registry record as it allowed investigators to extract and synchronize information from both the record book and the diabetes registry record. Furthermore, the total diabetes registry record in the clinic was numbered at 1048; however, it dates back to 1992 and was never updated. The 1048 records included many patients who had moved, died, or defaulted completely.

As the patient’s visit interval to the clinic may vary, to recruit based on the attendance of the patient, we needed to remove the duplicates while not excluding patients with longer follow-ups. The follow-up duration that was given to any diabetic patient in this clinic was between 1 week and 3 months; thus, 3 months was chosen as the duration of our study. Based on information from September 2015 to November 2015, we found that there was an average of 440 unique visits in a total of 1074 visits in 3 month, and based on our estimation, a recruitment of roughly every 5–6 patients would have been sufficient for our sample size requirement of 205 (based on 440 unique visits). In the end, we decided to recruit the first and then every 6th patient after who visited the NCDs clinic in Lundu Hospital, from every weekday from January 1 to March 30, 2016, synchronizing information from both record books of patients and diabetes registry records.

Inclusion criteria were as follows: patients older than 18 years, diagnosed as having Type 2 DM, not pregnant, and followed up for at least 1 year in the NCDs clinic. A total of 260 patients were recruited. Among them, 33 patients were found to be duplicated, 10 of them had only been followed up for less than 1 year in the NCDs clinic, and three had been transferred from other NCDs clinics, with poor documentation. Therefore, a final total of 214 were considered for further data analysis. Three qualified medical doctors who were also the investigators of this study were involved in the digitalization of the diabetes registry records. A digital data form is created for each entry with necessary information required to be filled in in order to proceed to prevent incomplete data collection.

### Data collection

Data collection was performed by retrieving data from the diabetes registry record and the patient’s record book. The first page of the records containing information regarding initial registration in the NCDs clinic, demographics, medical history, risk factors, the year of diagnosis, and BMI were obtained. The latest visit with documentation of BMI, biochemical results, and last date of biochemical tests were obtained.

### Therapeutic target achievements

Therapeutic target achievements for the three NCDs (DM, HPT, and DLP) were defined similarly to the latest national data, namely the National Diabetes Registry Report 2012 (NDR), defining a single target therapeutic range for DM, HPT, and DLP. The target therapeutic range for DM was defined as 6.5% or below, HPT was defined as ≤ 130 mmHg/90 mmHg and DLP target as low-density lipoprotein cholesterol (LDL-C) ≤ 2.6 mmol/L^15^. The target biochemical examination regular interval for HbA1c was ≤ 12 months^18^, while that of FLP was defined as ≤ 6 months^19^, following the latest Malaysian clinical practice guidelines during the conduction of the research.

### Statistical analysis

The collected data were analyzed using the Statistical Package for Social Science (SPSS for Windows program version 25; IBM, Armonk, New York, USA). Descriptive statistics were calculated for demographic information, therapeutic target achievement, and biochemical examination interval. The data obtained were further classified into dichotomous factors. Three separate logistic regression models were used to measure the odds ratio (OR) of the achievement of therapeutic control targets for DM, HPT, and DLP. A P*-*value of less than 0.05 was considered significant.

### Ethical considerations

This study protocol was approved by Medical Research and Ethics Committee (MREC) of the National Medical Research Register (NMRR) (approval number NMRR-14-1867-23844, issued on October 19, 2015). All methods were performed in accordance with the relevant guidelines and regulations. Permission to conduct the research in Hospital Lundu was given by MREC and director of Hospital Lundu. The requirement for informed written consent from each participant was waived by MREC, as this study only included information from existing medical records and do not involve interaction with patient or collection of identifiable private information. Each entry of sample data was assigned an identification number in place of names to ensure confidentiality.

## Results

Table 1 indicates the sociodemographic characteristics of the 214 patients. In total, 81 (37.9%) men and 133 (62.1%) women were included. The duration of follow-up in the NCDs clinic ranged from 1 to 33 years. The participants’ mean age was 58.7 (standard deviation [SD], 12.1). The greatest proportion (35.5%) of patients belonged to the Malay ethnicity, followed by Bidayuh at 26.6% and Iban at 19.6%. After combining different ethnicity into indigenous and non-indigenous people, 46.2% of the participants were grouped as indigenous populations. This study found that 87.9% of the patients had HPT, 70.6% had DLP, and 62.6% had all three diseases (DM, HPT, and DLP). Male smokers outnumbered female smokers by more than 10 times (25.9% vs. 1.5%). There were five patients (2.3%) with peripheral artery disease, four patients (1.9%) with coronary heart disease, three patients (1.4%) with cerebrovascular accidents, and two patients (0.9%) with renal failure. For biochemical monitoring, 81.3% of the patients had HbA1c performed yearly and 74.3% had semiannual FLP.

**Table 1:** Sociodemographic characteristics of patients (N = 214).

Characteristics	Male		Female		Total	
	N	(%)	N	(%)	N	(%)
Total	81	(37.9)	133	(62.1)	214	(100.0)
**Age**						
21–40	5	(6.2)	4	(3.0)	9	(4.2)
41–60	48	(59.3)	74	(55.6)	122	(57.0)
61–80	24	(29.6)	52	(39.1)	76	(35.5)
> 80	4	(4.9)	3	(2.3)	7	(3.3)
**Ethnicity**						
Indigenous population						(46.3)
Bidayuh	26	(32.1)	31	(23.3)	57	(26.7)
Iban	18	(22.2)	24	(18.0)	42	(19.6)
Non-Indigenous population						(53.7)
Malay	21	(25.9)	55	(41.4)	76	(35.5)
Chinese	15	(18.5)	21	(15.8)	36	(16.8)
Indian	1	(1.3)	2	(1.5)	3	(1.4)
**Currently smoking**						
No	60	(74.1)	131	(98.5)	191	(89.3)
Yes	21	(25.9)	2	(1.5)	23	(10.7)
**BMI (kg/m**^**2**^**)**						
< 30	59	(72.8)	95	(71.4)	154	(72.0)
≥ 30	22	(27.2)	38	(28.6)	60	(28.0)
**Comorbidities**						
HPT	69	(85.2)	119	(89.5)	188	(87.9)
DLP	58	(71.6)	93	(69.9)	151	(70.6)
All 3 diseases	50	(61.7)	84	(63.2)	134	(62.6)
**Biochemical monitoring**						
HbA1c yearly	62	(76.5)	112	(84.2)	174	(81.3)
Less frequent than yearly	19	(23.5)	21	(15.8)	40	(18.7)
FLP 6 monthly	59	(72.8)	100	(75.2)	159	(74.3)
Less frequent than 6 monthly	22	(27.2)	33	(24.8)	55	(25.7)

Table 2 describes the therapeutic target achievement for DM, HPT, and DLP. Table 3 describes the binary logistic regression analyses for the therapeutic target achievement for DM and DLP after adjustment.

**Table 2:** Therapeutic target achievement for DM, HPT, and DLP.

Characteristic	Achieved		
	N	N	(%)
DM	214	92	(43.0)
National DM average in 2012			(23.8)
HPT	188	58	(30.9)
National HPT average in 2012			(47.6)
DLP	151	54	(35.8)
National DLP average in 2012			(37.8)
Two or more diseases	205	63	(30.7)
All three diseases	134	9	(6.7)

**Table 3:** Adjusted odds ratio (AOR) and 95% confidence interval (CI) of factors for therapeutic target achievement.

Characteristic	DM	DLP	All
	AOR (95% CI)	AOR (95% CI)	AOR (95% CI)
**Sex**			
Female	1 (reference)	1 (reference)	1 (reference)
Male	0.87 (0.46–1.68)	1.68 (0.70–4.05)	4.26 (1.06–17.05)*
**Age**			
< Mean years	1 (reference)	1 (reference)	1 (reference)
≥ Mean years	2.50 (1.32–4.74)**	1.63 (0.70–3.81)	4.50 (0.93–21.73)
**Ethnicity**			
Non-indigenous population	1 (reference)	1 (reference)	1 (reference)
Indigenous population	1.31 (0.73–2.38)	2.31 (1.04–5.12)*	1.99 (0.50–7.86)
**Smoking habit**			
Non-smoker	1 (reference)	1 (reference)	1 (reference)
Smoker	1.03 (0.38–2.83)	0.74 (0.21–2.62)	–
**Duration of follow-up**			
≥ Mean years	1 (reference)	1 (reference)	1 (reference)
< Mean years	2.58 (1.35–4.96)**	0.50 (0.21–1.17)	2.94 (0.53–16.32)
**BMI**			
≥ 30	1 (reference)	1 (reference)	1 (reference)
< 30	1.96 (0.98–3.91)	3.32 (1.15–9.58)*	1.30 (0.23–7.38)
**HbA1c monitoring**			
Not yearly	1 (reference)	1 (reference)	1 ( reference reference)
At least yearly	2.30 (1.04–5.06)*	–	1.52 (0.13–17.23)
**FLP monitoring**			
Not every 6 months	1 (reference)	1 (reference)	1 (reference)
At least 6 monthly	–	0.9 (0.35–2.31)	1.21 (0.19–7.71)

*P < 0.05; **P < 0.01.

### Diabetes mellitus

The proportion of patients achieving the HbA1c target was 43.0% in this study, with a mean HbA1c of 7.7% and mean follow-up duration of 8.4 years (Table 2). Age (unadjusted odds ratio [UOR] 2.06, 95% confidence interval [CI] 1.19–3.56, *P* < 0.01), BMI (UOR 2.16, 95% CI 1.14–4.08, *P* < 0.05), and HbA1c yearly monitoring (UOR 2.30, 95% CI 1.08–4.89, *P* < 0.05) were found to have significant effects on achieving target therapy control for DM. Other factors such as sex, ethnicity, smoking habit, duration of follow-up, and FLP monitoring were not significantly associated with achievement of target therapy control for DM. DM patients with HbA1c yearly monitoring (adjusted odds ratio [AOR] 2.30, 95% CI 1.04–5.06, *P* < 0.05), age ≥ 58.7 (AOR 2.50, 95% CI 1.32–4.74, *P* < 0.01), and duration of DM follow–up < 8.4 years (AOR 2.58, 95% CI 1.35.4.96, *P* < 0.01) remained significant after adjusting for age, sex, ethnicity, current smoking habit, BMI, duration of DM follow-up, and HbA1c yearly monitoring.

### Hypertension

The proportion of patients with HPT in this study was 87.9% (N = 188), with a mean age of 60.4 years and follow-up duration of 10.5 years. Mean systolic BP was 138.5 mmHg and diastolic BP was 75.6 mmHg. Therapeutic target achievement for HPT in diabetic patients was 30.9% (Table 2).

None of the factors were found to be significantly associated with HPT therapeutic target achievement before as well as after adjustments for age, sex, ethnicity, smoker, BMI, and duration of HPT follow-up.

### Dyslipidemia

The proportion of patients with DLP was 70.6% (N = 151), with a mean age of 58.8 years and a follow-up duration of 5.8 years. The means of the lipid profile for total cholesterol (TC), triglyceride (TG), high-density lipoprotein cholesterol (HDL-C), and LDL-C were 4.99 mmol/L, 2.15 mmol/L, 1.27 mmol/L, and 2.80 mmol/L, respectively. Therapeutic target achievement for DLP in diabetic patients was 35.8% (Table 2).

For DLP, indigenous population ethnicity (UOR 3.01, 95% CI 1.45–6.21, *P* < 0.01) and BMI < 30 (UOR 3.03, 95% CI 1.23–7.45, *P* < 0.05) were found to be positively associated with DLP therapeutic target achievement. Indigenous population ethnicity (AOR 2.31, 95% CI 1.04–5.12, *P* < 0.05) and BMI < 30 (AOR 3.32, 95% CI 1.15–9.58, *P* < 0.05) continued to be significant factors for DLP therapeutic target achievement after adjustments for age, sex, ethnicity, smoker, BMI, duration of DLP follow-up, and FLP monitoring (Table 3).

### Two diseases

The proportion of patient with at least two NCD was 95.8% (N = 205). In this group of patient, 30.7% have at least two NCD achieving target therapeutic level.

### All three diseases

The proportion of patients with all three diseases was 62.6% (N = 134), with a mean age of 60.2 years and follow-up duration of 6.2 years.

For patients with all three diseases, only 6.7% achieved the therapeutic targets for all three diseases. No current smoker found in this group. No significant factors affecting therapeutic targets for all three diseases were found. However, after adjusting for age, sex, ethnicity, BMI, duration of follow-up, HbA1c yearly monitoring, and FLP monitoring, male sex (AOR 4.26, 95% CI 1.06–17.05, *P* < 0.05) showed an association with therapeutic target achievement four times higher than in females (Table 3).

## Discussion

This study is one of the first to highlight target therapeutic control achievement for DM as well as HPT and DLP control in diabetic patients among the indigenous population ethnicities of rural east Malaysia. The control of DM and DLP in diabetic patients was found to be at least equal or better in rural areas in Malaysia at the national level; however, control of HPT was worse. Those receiving HbA1c yearly monitoring were twice as likely to meet the target therapeutic achievement for DM.

Good diabetic control has favorable long-term macrovascular and microvascular outcomes^20^. In this study, HbA1c yearly monitoring was performed in 81.3% of patients, who were found to be twice as likely to achieve therapeutic targets of DM. We can only speculate that the association may be due to good vigilance and attention given to the disease by both the patient and healthcare provider. First, the general compliance of the patient; they did not miss the scheduled follow-ups so that HbA1c tests could be performed. Second, the HbA1c result assisted the doctor in recognizing the patient’s average control over the past 3 months, rather than relying on the fasting blood sugar that varies highly based on the activity of the patient in the past few hours. Both factors lead to better treatment compliance and an improved response to changes in the disease. Given its straightforward implementation of making such tests available to perform, the government should consider tightly enforcing yearly HbA1c monitoring to ensure better diabetic control outcomes.

Moreover, older age and shorter duration of DM were found to be two times more likely to contribute to achieving diabetic control. This is in contrast with the common idea that older age is associated with non-adherence to medication, due to factors such as isolation and cost-related non-adherence^21,22^. However, with Malaysia being a family centric culture and medical care being provided practically free of cost, older age may not lead to the same problems found in other countries. The finding of shorter durations of disease leading to better control of DM, on the other hand, was expected and corresponded well with the fact that diabetic control worsens over time^23^.

DM is known to be an independent risk factor for cardiovascular diseases^24^, and DM itself was found to have a magnifying effect on cardiovascular risk for other risk factors, such as DLP and HPT^25^. The mortality of diabetic patients also tends to be higher for coronary diseases^26^, making control of HPT and DLP in diabetic patients important. DLP control was found to be three times greater with BMI < 30 and two times greater among indigenous population ethnicities. The relationship between BMI and DLP has been studied extensively^27^, while the association between ethnicity and DLP control is unclear. A previous study from Malaysia reported that indigenous population Sarawakians had similar metabolic syndrome prevalence as other ethnicities, although the components of the metabolic syndrome were numerous, and a specific identification could not be made^28^. Although another study found that east Malaysians engage in lesser physical activity than Malays and Chinese, the ethnicities were not grouped further in the east Malaysian sample^29^. In the same study, however, higher physical activity was found to be associated with rural communities, which may partially explain the better DLP control in the indigenous population group in this study^29^.

A dedicated description of DM, HPT, and DLP control in diabetic patients in rural east Malaysia was not previously available. Using the same targets as the NDR, this study found the DM and DLP control of diabetic patients in Lundu to be at least equal to national levels but was poor for HPT control. The target achievement of HbA1c at 6.5% or lower was 43.0%, which was significantly higher than the national level of 23.8% and any of the West Malaysian states (14.9–31.1%)^15^, as well as that of the Sarawak State as a whole, 39.1%^15^. The control of DLP in our study was 35.8%, roughly equal to 37.8% in the NDR^15^, while the target blood pressure achieved in our study was 30.9%, which is lower than the national mean of 47.6% in 2012^15^. A major difference between the figures for Lundu and the national figures may be due to the predominant indigenous population demographic in Lundu. Indigenous populations were traditionally rice farmers/fishermen, and those who remain in rural areas mostly still engage in physically demanding agricultural work^30^, and hence indirectly lead a healthier lifestyle. A study from Saudi Arabia supports the findings of this study, noting that rural regions are associated with better control of DM than urban regions^31^. The finding of worse HPT control in rural areas also reinforces the findings of a study in west Malaysia, which found that membership in the rural population is a risk factor for poor control of HPT^32^. A higher emphasis should be placed on HPT during health promotion activities and education programs in rural Malaysia.

As for the two-thirds of patient having all three diseases in this study, it was found that male sex was associated with achieving good control of all three diseases. Sex-specific pathophysiological differences in metabolic syndromes of which DM, HPT, and DLP were part of have been documented^33^, but direct link with control in terms of target therapeutic achievement has not been established. Also, we speculate that influenced by a more traditional male-dominant family and society in the rural area where resources are limited, a more frequent healthcare appointment to optimise control of multiple comorbidities may be out of reach for some females. A bigger study focusing on sociodemographic, sex and compliance to follow-up is needed to fully explore the association.

The results of this study provide suggestions for future research directions. This study found that indigenous population ethnicity was significant in controlling DLP, perhaps due to a genetic predisposition or cultural lifestyle, suggesting that future research that includes east Malaysians should consider ethnicity as a factor in the analysis.

There are limitations to this cross-sectional study. Other important sociodemographic factors such as education level, income, and distance from healthcare centers were not included in this study. The target population was limited to patients with DM. The sampling method may have indirectly excluded patients with poor diabetic control, as patients who defaulted treatment for more than 3 months would not be included in the sampling period. This study was conducted only in one of the twenty-eight rural districts in Sarawak, east Malaysia.

## Conclusions

In conclusion, this study revealed that rural east Malaysia may experience a different pattern of NCDs control when compared to the rest of Malaysia. This provides useful data for local healthcare officials to plan health promotion strategies. Ensuring HbA1c monitoring may be a useful tool in achieving target therapy control for DM. Accessibility to laboratory tests and enforcement of HbA1c monitoring in diabetic patients must be ensured in all healthcare centers for better control of diabetes. The patients’ ethnicity needs to be considered while examining dyslipidemia in east Malaysia.

## Data Availability

The author has ownership of the data. However, as the research was conducted in government hospital, when sharing data with personnel nonaffiliated with Ministry of Health Malaysia, it is advisable to request and obtain permission from the Institution for Medical Research Malaysia, the NMRR & MREC Secretariat for researchers who meet the criteria for access. Researchers who would like to access to the data must contact the institution for Medical Research Malaysia, the NMRR & MREC Secretariat at Institute for Medical Research, Jalan Pahang, 50588 Kuala Lumpur, Malaysia. Tel: +603-26162666 Fax: +603-26939335, E-mail: Portal@imr.gov.my.

## Abbreviations

NCD: Non-communicable disease
DM: Diabetes mellitus
HPT: Hypertension
DLP: Dyslipidemia
AOR: Adjusted odds ratio
CI: Confidence interval
WHO: World Health Organisation
UHC: Universal health coverage
BMI: Body mass index
FLP: Fasting lipid profile
NDR: National diabetes registry report
NHMS: National health and morbidity survey
MREC: Medical Research and Ethics Committee
NMRR: National Medical Research Register
SD: Standard deviation
UOR: Unadjusted odds ratio
LDL-C: Low-density lipoprotein cholesterol
HDL-C: High-density lipoprotein cholesterol
TC: Total cholesterol
TG: Triglycerides
